# Exploring community participation in project design: application of the community conversation approach to improve maternal and newborn health in Zambia

**DOI:** 10.1186/s12889-017-4187-x

**Published:** 2017-03-23

**Authors:** Wilbroad Mutale, Chisala Masoso, Bisalom Mwanza, Cindy Chirwa, Lasidah Mwaba, Zumbe Siwale, Barbara Lamisa, Dennis Musatwe, Roma Chilengi

**Affiliations:** 1 Department of Public Health, University of Zambia School of Medicine, Lusaka, Zambia; 2 Centre for Infectious Disease Research in Zambia (CIDRZ), Lusaka, Zambia

**Keywords:** Community conversation (CC), Antenatal (ANC), Contraceptives, Family planning, Maternal health, Newborn health

## Abstract

**Background:**

The United Nations Development Programme (UNDP) has adopted an approach entitled Community Conversation (CC) to improve community engagement in addressing health challenges. CCs are based on Paulo Freire’s transformative communication approach, in which communities pose problems and critically examine their everyday life experiences through discussion. We adopted this approach to engage communities in maternal and newborn health discussions in three rural districts of Zambia, with the aim of developing community-generated interventions.

**Methods:**

Sixty (60) CCs were held in three target districts, covering a total of 20 health facilities. Communities were purposively selected in each district to capture a range of rural and peri-urban areas at varying distances from health facilities. Conversations were held four times in each community between May and September 2014. All conversations were digitally recorded and later transcribed. NVivo version 10 was used for data analysis.

**Results and Discussion:**

The major barriers to accessing maternal health services included geography, limited infrastructure, lack of knowledge, shortage of human resources and essential commodities, and insufficient involvement of male partners. From the demand side, a lack of information and misconceptions, and, from the supply side, inadequately trained health workers with poor attitudes, negatively affected access to maternal health services in target districts either directly or indirectly. At least 17 of 20 communities suggested solutions to these challenges, including targeted community sensitisation on the importance of safe motherhood, family planning and prevention of teenage pregnancy. Community members and key stakeholders committed time and resources to address these challenges with minimal external support.

**Conclusion:**

We successfully applied the CC approach to explore maternal health challenges in three rural districts of Zambia. CCs functioned as an advocacy platform to facilitate direct engagement with key decision makers within the community and to align priorities while incorporating community views. There was a general lack of knowledge about safe motherhood and family planning in all three districts. However, other problems were unique to health facilities, demonstrating the need for tailored interventions.

## Background

To improve community engagement in the fight against HIV, the United Nations Development Programme (UNDP) has adopted an approach known as ‘Community Conversation’ (CC) [1]. The term “Community Conversation” is used to describe community discussions among local people that address a common problem and are guided by trained facilitators. The aim of the conversation is to stimulate and support critical thinking among participants to identify solutions to problems affecting their community. This approach has been used elsewhere to address various issues affecting diverse communities [2]. Some of these issues were related to health, such as mental health or health inequalities, while others have used CCs to address non-health-related issues such as youth unemployment and early childhood education [3].

CCs are generally considered to be a new and unique type of intervention that is distinct from focus groups, as rather than simply discussing an issue, CCs have a clear agenda of generating action plans and supporting the community to think critically about their problems and to find local solutions. Generally, focus groups are considered to be more research oriented, with the aim of collecting information about social relations and understanding phenomena. In contrast, CCs are explicitly aimed at changing participants’ world views and their conceptions of what is possible [2, 3].

Advocated by the education theorist and social activist Paulo Freire, the rationale behind CC is that critical thinking by marginalised groups can often constitute a key pre-condition to health-enhancing individual change and social action. Freire’s ‘transformative communication’ approach has highlighted processes through which subjects pose problems and critically examine everyday life experiences through discussion. While similar community approaches have been reported, the specific application of CC is uncommon in resource-poor settings and is scarcely represented in the peer-reviewed literature [1]. In one example in rural Zimbabwe, CC was used to contribute to local HIV competence by enabling community members to brainstorm concrete action plans for responding to HIV and by providing a forum to develop a sense of common purpose [1]. CCs also helped the participants overcome fear, denial and passivity. However, the authors cautioned that while this methodology could empower communities, it may be hindered by the broader social and political context [1].

While the CC approach has been useful in community mobilisation and empowerment, its application has been limited to responses to HIV in sub-Saharan Africa [1]. Other health problems such as maternal and child health certainly stand to benefit through better community engagement approaches [1].

Zambia was unable to achieve Millennium Development Goals (MDG) 4 & 5 due to slow progress [4]. Although infant and maternal mortality is largely preventable, neonatal and maternal health in Zambia remain poor, with current statistics for neonatal and maternal mortality at 24 per 1000 and 389 per 100,000 live births, respectively [4]. As with many other health statistics, rural communities have consistently worse outcomes than their urban counterparts [4]. Better health systems and community engagement have been identified as crucial ingredients to attaining MDG 4 & 5, particularly in rural areas [4, 5].

In this paper, we report our experiences and outcomes of applying the CC approach to engage communities around maternal and newborn health in three rural districts of Zambia. This approach is a departure from the often donor-driven top-down models to a more participatory method, which is known to empower communities and make them accountable for their chosen deliverables.

## Methods

### Study setting

Zambia has predominantly free health care services, with only a few private providers in urban areas. Government policy encourages women to attend at least 4 antenatal care (ANC) visits during a single pregnancy and to deliver in health facilities attended by qualified health personnel. Current statistics indicate that 66% of births occur in a health facility, while 31% occur at home; births in urban areas are more likely to be at a health facility (89%) than births in rural areas (56%). Socio-cultural and structural factors, as well as a lack of knowledge of maternal and newborn care, act as barriers to seeking health care among families, particularly those in rural areas [4].

### The better health outcome through mentorship and assessment project: BHOMA II

The BHOMA II project was a community intervention funded by Comic Relief to address maternal and newborn health in three districts in Zambia. BHOMA II was a follow-on to BHOMA I, which focused mainly on strengthening the health system in the same districts. CCs were held as part of the baseline activities of BHOMA II to facilitate the design of appropriate interventions while accounting for community concerns. This paper focuses on this baseline assessment. CCs were expected to be repeated again after 12 months of intervention.

### Community conversations

CCs were used to collect information in relation to maternal and newborn health in three rural districts of Zambia. The goal of the CC was to establish baseline challenges to address during the 12-month intervention period. After 12 months, CCs were designed to be repeated to assess the extent to which the interventions had addressed the baseline challenges. The questions were tailored to elicit challenges to maternal and newborn health and to establish actual and latent local responses that capitalised on strengths existing within the community. CC facilitators were trained community residents who were familiar with the local context and the languages spoken in target communities.

### Target communities

Sixty (60) CCs were held in three target districts. Communities served by 10 health facilities in Chongwe, 4 in Luangwa and 6 in Kafue were purposively selected to participate in CCs. Purposive sampling within each district was performed to ensure representation of rural and peri-urban areas that were pre-determined varying distances from a health facility. Three of the sites were classified as peri-urban, while the remaining 57 were considered rural. Distance from health facilities was used as one of the criteria for community selection to capture a representative spectrum of the challenges facing communities in maternal and child health.

### Data collection process

Four sessions were held in each community between May and September 2014, consisting of three CCs and one validation session that followed standard procedures [2]. The first CC session focused on exploring issues that affected communities in relation to family planning, ANC attendance, labour and postnatal services. The second session explored in detail the specific themes and challenges identified in the first session. Session three summarised the issues raised and allowed participants to suggest related solutions and community contributions to enacting these solutions. Session four was a validation session in which key stakeholders such as district and health centre staff, traditional leaders and politicians were informed of the issues discovered in the preceding three CCs and invited to provide suggestions on how these issues could be best addressed. The conversations lasted approximately 2 h each, and both men and women participated in each conversation. The questions focused on maternal and child health and explored the following themes:

- General concerns regarding reproductive health in the community;
- Challenges in accessing family planning services;
- Challenges in attending ANC;
- Challenges in facility-based delivery;
- Risk factors associated with pregnancies; and
- Perceived causes of maternal and neonatal mortality within the community.

Within each theme, we explored some of the perceived benefits of health care service utilisation and the underlying causes for failure to use these services. In addition, community responses to the challenges were discussed, which led to summary action plans with clear roles for the community and the project. We used a fishbone diagram to graphically summarise the linkages between challenges and outcomes.

### Data management and analysis

Interviews were recorded, transcribed and coded inductively. Three research assistants trained in qualitative methods transcribed the interviews. Transcripts were cleaned and exported to NVivo 10 (QSR International; Melbourne, Australia) for analysis. Two of the authors (WM and RC) reviewed the interview transcripts, validated the pre-determined themes and identified additional themes and subthemes that emerged. Two researchers coded the data, and Cohen’s Kappa statistic was used to assess inter-coder reliability [6]. Data were organised by pre-determined themes. These formed the basis for broader themes, which were further sub-categorised to increase the explanatory ability of the data.

### Selection of projects for consideration

To ensure that sustainable projects emerged from the data collected, stringent criteria were set a priori, and only proposals that met these criteria were selected for consideration. These criteria included the following:

1. Identification of at least 5 possible interventions, each with measurable indicators.
2. Proposal of how the community would take ownership and sustain the project.
3. Clear description of community contribution and expected project support.
4. Feasibility of implementation with demonstrable results anticipated within 18 months.

### Ethical consideration

Ethical approval was granted by the University of Zambia Bioethics Committee. Participants were informed about the purpose of the conversations. Verbal consent was obtained from each participant.

## Results

The results are divided into two sections. Section one describes the common problems and challenges faced by community members in accessing maternal health services. Section two summarises the intervention suggested by the community to address these challenges in partnership with the project.

The major problems identified by community members were geographical access, limitations in infrastructure, lack of knowledge, shortage of human resources and lack of commodities. These barriers were reported to limit the utilisation of maternal health services either directly or indirectly.

### Demographic characteristics of participants

Overall, 605 people participated in the CCs, 50.4% of whom were female. Table 1. provides a summary of the attendance and participation in the four sessions for each community.

**Table 1:** Participants: community conversations

Step	Date of Implementation	District	Health facility	No. of females	No. of males	Total
Step 1	Jun-14	Kafue	Kambale	12	16	30
		Kafue	Kazimva	23	22	45
		Chongwe	Lwiimba	9	11	20
		Chongwe	Mwalumina	13	7	20
		Luangwa	Luangwa Boma	12	13	25
Step 2	Jul-14	Kafue	Railways	24	21	45
		Kafue	Kris Katumba	17	8	25
		Chongwe	Kasisi	13	17	30
		Chongwe	Zasti	11	14	25
		Luangwa	Chitope	10	15	25
Step 3	Sep-14	Kafue	Chilanga	24	26	50
		Chongwe	Mpango	18	22	40
		Chongwe	Chinyunyu	21	19	40
		Chongwe	Nyangwena	16	14	30
		Luangwa	Mphuka	9	16	25
Step 4	Oct-14	Kafue	Mwembeshi	17	13	30
		Chongwe	Rufunsa	9	11	20
		Chongwe	Chainda	22	18	40
		Chongwe	Ngwerere HP	8	7	15
		Luangwa	Kasinsa	10	15	25
			Total	298	305	605

### Geographical access

Geographical access to health services was a major challenge in most rural communities. Long distances to health facilities meant that members of the community had to spend many hours to reach a facility. Communities described travel time and distance as affecting pregnant women because of the associated risks, especially if pregnant women travelled alone or while in labour. The problem was exacerbated by the inadequacy of health outreach programmes and the absolute lack of reliable public transport services in most areas. Participants identified unreliable or unavailable ambulance services as a barrier to routine health-seeking behaviour as well as a real danger, particularly for pregnant and labouring women, in the case of emergencies. Transportation limitations were noted as contributing to the poor rates of antenatal attendance, health facility deliveries and postnatal attendance, as expressed by some participants in CCs:

“Because labour starts all of a sudden, we see women giving birth on the way, as the labour ward is very far. If we had anything nearer, we wouldn’t have been experiencing such challenges.”

Male participant, Luangwa

“The other reason that women give birth from homes and on the way to the hospital is because we do not have transport. The ambulance that has been provided for Luangwa does not get to us, we just see it from a distance.”

Female participant, Luangwa

“Yes, the problem is really big here, as the majority of the villages are very far away from the health facility … How do you honestly expect a pregnant woman in labour to use an oxcart with the rough roads? I have my own experience with transport; my own wife was due for delivery, and she asked me to take her to the centre; due to the unavailability of transport, I straddled her onto my farm tractor and started heading to the clinic. On our way, the child dropped from her as I sped on the bad road. And when I stopped, I found that the child was still attached to the mother, and I managed to wrap it, and we managed to get to the centre.”

Male participant, Kafue

### Infrastructural challenges

During conversations, community members in some sites expressed concern about the limited space in health facilities, especially for those who required supportive maternal health services. Because there were no ambulance services in rural areas, expectant mothers living far away from health facilities preferred to stay close to the health facility when the delivery date was close. This indicated the need for a mother’s shelter close to delivery centres with essential facilities such as water and toilets. However, most health facilities did not have this type of shelter in place, making it difficult for women who lived in remote communities to deliver in health facilities.

“Pregnant women need to move close to the clinic before it is too late … if there was a shelter, it could have been much easier for them to sleep and even prepare some food for themselves.”

Male participant, Kafue

A lack of privacy was identified as a barrier to delivery in health facilities. Some delivery centres were so small that women in labour could not be afforded privacy. Participants noted that this lack of privacy made some expectant mothers reluctant to deliver in a health facility, and they thus preferred to deliver at home, where their comfort and privacy may be better ensured.

“ … sometimes women are afraid because the facility is small and the people will hear you when you start crying. Sometimes men are passing [by], so it is embarrassing. There is also no toilet, no shower, It is embarrassing for everyone to be looking at you. To avoid this embarrassment, it [is] better to deliver from home.”

Female participant, Chongwe

### Availability of essential commodities

CC participants noted that pregnant women were expected to bring supplies needed to deliver in a facility, including surgical gloves, a bucket for water, razor blades, cord clamps, and methylated spirit. Many community members felt that women who were unable to meet this expectation often delivered at home to avoid the embarrassment.

“I had a case like that. One mother came at night and did not have anything for a safe delivery. No gloves, no razor, no blanket; she was unprepared. So most women decline [to go] to the clinic when they realise they are not prepared.”

Traditional birth attendant, Chongwe

“The problem is that the delivery requirements are expensive. So many who cannot afford [them] stay home.”

Headman, Kafue

Another service affected by scarce commodities was family planning. Participants stated that long-acting contraceptives were preferred by most women but that they were often not in stock at health facilities. It is not unusual for the supply of contraceptives to run out at health facilities, making couples vulnerable to pregnancy. As an alternative, some couples are advised to buy from private pharmacies.

“I want to talk about Jadelle [a long-acting reversible contraceptive implant]; it is not in stock most of the time. A person could want to change from oral contraceptive because it causes side effects, but when you come here, you are only told that it is not available.”

Female participant, Kafue

“There are times that when a woman comes to access family planning, it is discovered that there is nothing available, and so they are advised to go and buy from a private chemist. But that woman has no money, and she will end up going back home and conceiving. So we are requesting that these drugs be readily available at all times.”

Female participant, Chongwe

### Human resource shortages

Participants identified inadequate human resources, at both the community and health facility level, as contributing to poor health services, particularly in the most rural areas. The CCs revealed that most health facilities were managed by one trained health worker. Although the facilities were supported by community volunteers such as traditional birth attendants (TBAs) and Safe Motherhood Action Groups (SMAGs), the few health workers were often overworked and functioned as the sole provider in busy facilities:

“We have also noticed that sometimes only one nurse is on duty, and she cannot handle all the cases. So some mothers simply end up going home. When these women have to wait long, they get discouraged.”

Male participant, Chongwe

**“**I think a lack of man power at the clinic is a major problem. When you go to the clinic, you find one doctor, and when an emergency happens, he is going to rush for the emergency, leaving you waiting; as you know, he can’t handle two cases at once.”

Female participant, Luangwa

Participants noted that in many communities, the delivery attendants in the health facility were often TBAs, who were called on to support the trained health worker.

“ …. the problem is that even if they deliver at the clinic, they are helped to deliver by the TBAs, not the nurses.”

Male participant, Kafue

Furthermore, because of few health workers per facility, services such as ANC outreach services were understaffed and inconsistent, which led many women to register late. One participant explained:

“For those who come from afar, they wait for the nurses to go there for outreach services because they cannot manage to come here. It’s very far. At times the nurse doesn’t go to the monthly outreach. So on the month that the nurse doesn’t go for the meeting, [mothers] don’t register until the nurses go to the outreach. That is why others register when they are 6 or 7 months pregnant.”

Female participant, Chongwe

Some participants noted that even if health workers are present, the community may not want them to conduct deliveries, particularly if they are male. Some male participants felt strongly about this and discouraged their partners from delivering at heath facilities where the only health worker was male.

“The problem is that the clinic is understaffed; the clinical officer is the one always on duty … and the clinical officer is our friend. So the mothers don’t want to be handled by the person who happens to know them. They prefer someone they totally don’t know or a fellow woman.”

Male participant, Chongwe

The lack of human resources was compounded by the limitations in the scope of work of the available health workers. Participants reported that some of the nurses at health facilities were not trained as midwives, and some of them lacked the skills to deliver babies.

“One of the problems that I have seen is that some of the nurses don’t know how to help women deliver. They are just nurses, but they are not trained to deliver. So we only have one nurse who knows how to deliver [babies from] pregnant mothers.”

Female participant, Kafue

The presence of a trained health worker did not necessarily translate into good services. Health workers’ attitudes were identified as an important factor that could negatively affect women seeking access to maternal health services. Across the three districts, community members were concerned about poor behaviour among health workers and poor attitudes towards patients, as illustrated by the quotes below. Some women gave personal testimonies of being scolded or chased away from centres for minor misunderstandings.

“Some women do not go to the clinic because they are more comfortable in the hands of the TBA than at the clinic. They fear being shouted at and sometimes insulted during delivery.”

Male participant, Chongwe

“Sometimes some women say that they can’t go for fear of being rebuked for being dirty by some nurses. Some are even rebuked for coming with unclean children or wearing the same clothes all the time.”

Female participant, Luangwa

“I also just wanted to echo the sentiments expressed by the previous speaker that women would rather give birth from home for fear of being shouted at here at the health centre by staff for not having all the requirements needed for labour. And so I would like to urge the nurses to be compassionate and understanding; it is not everyone that comes from a family that can afford what is needed.”

Female participant, Kafue

### Knowledge gap and misconceptions

Lack of information was also a major contributor to the low utilisation of maternal health services. Together with traditional cultural beliefs and practices, poor access to accurate health information was noted as a driver of major misconceptions. A general lack of information and misunderstanding were noted, particularly surrounding family planning services and home delivery. Most people were aware of family planning options, but they were either not willing to use them or had misconceptions that led to a poor uptake.

Male partners’ role in decisions regarding reproductive health, such as whether to use family planning, was identified as a barrier for women, who were often targeted when they were alone by health workers with family planning services. Men had misconceptions about modern contraceptive methods, linking these methods to promiscuity, cancer and infertility. Some women similarly endorsed these misconceptions and discouraged each other from using contraceptives.

“I used the pills at first and had side effects, and then I got injectables. But they too gave me problems, and so I reverted to pills. But the problem is that I can’t conceive now when I want to.”

Female participant, Kafue

“We know for a fact that when a woman takes these tablets for a long time, she fails to conceive. These things block her completely with little or no chance of ever conceiving.”

Male participant, Kafue

Some women were using contraceptives without the knowledge of their partner, and they were forced to hide this use for fear of stigmatisation, as explained by some women:

“We hide [our use of] family planning, [because] we fear arguments in the home and the little children in the home, so we hide [them] so that they are not found.”

Female participant, Kafue

“I also found the same problem of my wife hiding the pills from me when we had agreed to stop using family planning methods, having spaced our previous children; but I was surprised that she was still taking the pills secretly.”

Male participant, Luangwa

Some participants favoured using condoms, but others viewed condoms as a method for use with extra-marital sexual relationships and not for use with stable partners. This association of condoms with promiscuity or infidelity caused stigmatisation of their use by married couples.

“I think that if a couple starts using condoms in the house, it means that there is one or both of you who is promiscuous and [that] you don’t trust each other”

Male participant, Kafue

“ … the other thing is that churches don’t allow church members to use family planning, for example, Catholics.”

Male participant, Chongwe

Participants were divided as to the role of contraceptives for teenagers who were still in school. While they acknowledged that teenage pregnancies were problematic, most parents refused to allow their children to access modern contraceptives, fearing that they would lead to promiscuity, sexually transmitted diseases such as HIV, and pregnancy complications.

“I want to find out at what age a woman is supposed to access family planning. I say so because some women are allowing their children to start contraceptives so that they finish their education, and I think that is one reason that is causing all of these pregnancy complications.”

Male participant, Chongwe

“If you allow them [young people] to use family planning methods, that means they will have freedom and they will be having sex freely without even a worry; let’s just teach them what’s bad so that even when they are doing it, they are scared of the consequences.”

Male participant, Kafue

### Summary of major problems across all study sites

**Fig. 1:**
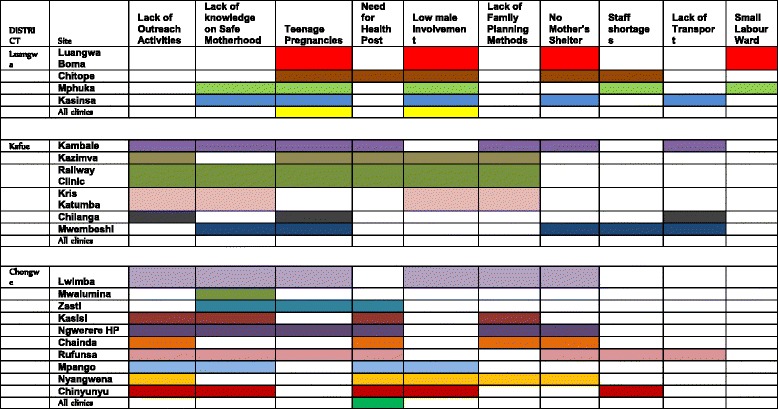
Community conversation - Summary of priority problems for target health facilities

**Fig. 2:**
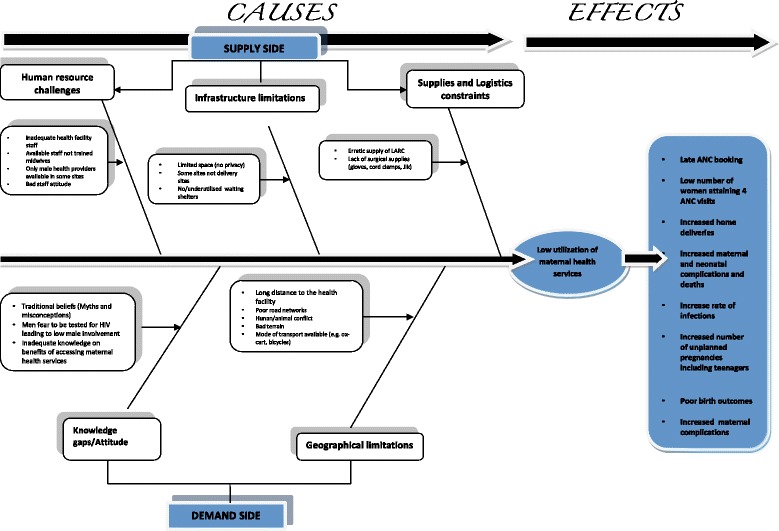
Fishbone analysis

Figure 1 provides a summary of the major challenges identified across all study sites. Many priorities were similar across the three districts. However, some differences were identified at the community/facility level. Lack of knowledge of safe motherhood practices, prevention of teenage pregnancy and low male involvement in family planning were cited in all three target districts. In Kafue and Chongwe, the need for outreach services was among the priorities identified. This was not a main priority in Luangwa district, where instead, mothers’ shelters and larger labour wards were the priorities (Fig. 1). The major challenges and consequences are depicted in the fishbone analysis (Fig. 2).

Community members discussed possible solutions to the challenges they faced and proposed interventions, including the contributions that community members could make and what would be required as support from the project (Table 2). Most of the proposed interventions targeted community sensitisation on the importance of safe motherhood, family planning and teenage pregnancy prevention (17/20). SMAGs were the main and preferred providers of sensitisation within communities. In terms of contribution, the community was responsible for providing volunteers, while the project was asked to train community health workers and provide information, education and communication materials. On the suggestion of two communities, the project procured motorcycle ambulances to facilitate transport problems. For this intervention, community members agreed to maintain the motorcycle ambulances and generate their own funds to meet the fuel and maintenance costs. Other facility-specific interventions and contributions are summarised in Table 2.

**Table 2:** Consolidated CCs summary

Site	Proposed interventions	Community Contributions	Project contribution
Lwimba	Build outreach post at Ndapula (11 km) and Lukoshi (15 km).construction of mothers shelter at the clinic	Support outreach health services. (NHCs, CHWs, TBAs).Provide sand, bricks, stones and labour towards the construction of mothers shelter	Material support toward construction of mother’s shelter (Not more than allocated amount to site)
Mwalumina	Construction of bathroom for mothers at the health centre	Provide bricks, sand stones and labour	Provide building materials for incomplete mother’s shelter
ZASTI	Community Sensitisations on safe motherhood issues	-Conduct continuous health education on safe motherhood issues during community meetings and drama performances	Sensitization on information of SMH
Kasisi	Community sensitisation on importance of safe motherhood health services	Form community groups in each zone for sensitization. Form drama group. Sensitization through door to door, vilage meetings and outreach services by CHWs, TBAs and NHCs	Sensitization on information of SMH
Ngwerere HP	Conduct community sensitization through community meetings, churches, door to door and Drama performances. Community members to also form drama group	Conduct Health Education on importance of ANC, HIV testing, Clinic delivery, postnatal care and family planning with emphasis on long term contraception during village meetings, farm meetings and NHCs meetings. Conduct drama performances	Cooperatives approach (ride on existing cooperative initiatives and include sensitization on SMH)
	Sensitization on dangers of teenage pregnancies in schools and communities through drama performances. Conduct health education in communities and schools. Sensitization and counseling of parents at community level on benefits of FP to teenagers and dangers of Teenage pregnancies	Drama groups to perform in schools and market places to sensitize teenagers on the dangers of abortions, HIV and importance of contraception.	Engage with SFH to provide training and LARC for use at clinic
Chainda	Carry out Drama performances in community on importance of males to support their wives. Use village headmen during community meetings to allow TBAs, NHCs CHWs provide health education and carry out community sensitization on importance of male involvement, and the benefits of utilization of ANC, facility delivery and family planning services.- equip knowledge to headmen and NHCs by carrying out trainings and skills on issues of safe motherhood in order to assist in dissemination of right information in communities	provide local drama group to carry out drama performances in all the zones.- Support the mobilization of people for the drama performances through NHCs, Headmen and health centre staff. HCC and health staff to identify community leaders and members to be trained on safe motherhood. HCC together with health staff to plan and monitor action schedule for the activities. HCC and heath staff to arrange a meeting with apostolic church leaders in chimbali area on issues of health in order to allow their members access health services.	Purchase marquee and put slab
	HCC to be holding meetings with health center staff to discuss best way forward to avoid misunderstandings.	HCC through the HCC chairperson to provide platform for dialogue on behalf of the community. TBAs to continue assisting the health staff in providing knowledge to mothers for clinic deliveries, and early ANC bookings.	
Rufunsa	To construct mothers shelter at the clinic for use by expectant mothers and their relatives	Mold bricks, provide sand,stones and Labour toward s the construction of the mothers shelter. Prepare the BOQ for other required building materials such as Cement, planks, roofing sheets,etc.	Material support toward construction of mother’s shelter (and provide sensitization in SMH to expectant mothers)
Mpango	Conduct community sensitization on safe motherhood issues	Sensitize community members through village meetings and NHCs. Conduct Drama performances	Sensitization in SMH issues
	Conduct community sensitization on importance of Birth preparedness and Male involvement	Teach community members through village meetings and NHCs on importance of birth preparedness and male involvement. Conduct Drama performances	
Nyangwena	Carry out drama performances in community on importance of males to support their wives	provide local drama group. Mobilize people for the performances through NHCs, Headmen and health centre staff.	Provide a marquee at site for venue for health talks
Chinyunyu	Community sensitization on importance of male involvement in safe mother hood issues	NHCs, CHWs, TBAs and community leaders to sensitize males during village meetings as well as drama performances	Provide a marquee at site for venue for health talks
KAMBALE	To conduct sensitisation in safe motherhood	Community mobilization, formation of drama group	Women’s groups (ride on existing social groupings; provide logistics for social activities)
	To train and support SMAGs	Volunteers [men and women] to be trained in SMAGS	
	To train and support youth friendly health services	Youths to be trained as peer educators	Women/youth groups (ride on existing social groupings; provide logistics for social activities)
KAZIMVA	To form Safe motherhood action groups. Sensitisation of the communityPromote male involvement in SMH activities	Commitment and volunteerism in terms of enrolling in SMAGs and being active, sanitization on safe motherhood	Provide a marquee at site for venue for health talks
	To train FP providers in long acting methods,	Training venue	Engage with SFH to provide training and LARC for use at clinic
	Sensitisation of community on FP, train CBDs, SMAGs	TBAs, CBDs, SMAGs to conduct sensitisation,	
RAILWAY CLINIC	To sensitise the community on safe motherhood activities, To form, train and support SMAGS	Volunteers [men and women] to be trained in SMAGS	Women’s groups (ride on existing social groupings; provide logistics for social activities)
	To form,train and support youths in YFHS	Peer educators, staff, teachers to be trained, training venue	Women/youth groups (ride on existing social groupings; provide logistics for social activities)
	To sensitise the community on safe motherhood activities and neontatal care	Community mobilization, Formation of drama group	Women’s groups (ride on existing social groupings; provide logistics for social activities)
KRIS KATUMBA	Mass sensitisation through local leaders, drama performances	Volunteers, drama groups	Women’s groups (ride on existing social groupings; provide logistics for social activities)
	Forming and training of Safe motherhood action groups, CBDs	Provide volunteers for training and the venue	Women’s groups (ride on existing social groupings; provide logistics for social activities)
	To provide adequate amounts of the FP methods	Sensitisation of the mothers o the available methods	Engage with SFH to provide training and LARC for use at clinic
CHILANGA	To conduct regular outreach in distant zones, To include SMH activities such as ANC,PNC,FP during outreach	Provide space for outreach., Mobilise communities during outreach services	Provide Portable Tents, couch, linen
MWEMBESHI	Create mass awareness through community sensitisation involving local leaders, SMAGs, TBAs, CBDs, NHCs and other social advocates, Promote male involvement in all SMH activities	Volunteers in form of SMAGs, TBAs, CBDs, NHCs, traditional, Church leaders etc., Community mobilisation, Use males as role models	Cooperatives approach (ride on existing cooperative initiatives and include sensitization on SMH)
Luangwa Boma	Strengthen community health education and male involvement.	SMAGS to conduct door to door HE and follow up all males that don’t escort their wives for ANC on a monthly basis.	Provide a marquee at site for venue for health talks
		Headmen to prepare a schedule of CC/meetings which will be followed by all.	
	Community meetings/conversations	Headmen & HC to identify and invite stakeholders to disseminate the RHS key messages.	
	Involving churches to disseminate Reproductive Health key messages.		
Chitope	Massive community sensitization	SMAGs, CHWs and TBAs to intensify community sensitization through door to door visitations.	Procure motorbike ambulance
	Construction of Mangelengele community health post.	Community to provide sand, stones and mould bricks.	
Mphuka	Construction of a Labour ward at the health facility	25% community contributions through moulding of blocks and provision of sand and stones	Procure bicycle ambulance
	Massive sensitizations by Safe motherhood Action Groups (SMAGs) on birth plans.	The area councilor to spearhead for the application of Constituency Development Fund	
	The Community to lobby to DMO to send a female midwife or Nurse to the facility	HC to provide Birth plans to the SMAGS/TBAs to be used during door to door visitations.	
		Full TBA Training to be conducted.	
Kasinsa	Intensify community sensitizations by Safe Motherhood Action groups, (SMAGs) Traditional birth attendants, CHWs, NHC Chairpersons and the headmen.	SMAGs to come up with an action plan of educating women on Birth Plan and continue with door to door visitations.	Procure motorbike ambulance
	Construction of a Mothers’ shelter at the health facility.	25% community contributions through moulding of blocks and provision of sand and stones.	
	Lobby for the purchase of Motorbike ambulances to help on transport.	The Health centre, HCC and the Councilors to work together and apply for Community Development Fund (CDF).	

## Discussion

Community conversations have been shown to empower communities by allowing them to take control of identifying and addressing their own challenges [1, 2]. Through a series of facilitated meetings, community problems and priorities were identified and solutions were proposed.

In our baseline CCs, the major problems identified were geographical access, infrastructure limitations, lack of knowledge of safe motherhood practices, inadequate human resources, and lack of commodities. These problems were discussed in-depth to determine the underlying causes as well as interventions aimed to address them. Community members discussed proposed solutions, focusing on the practicality of the solution and capacity of the community to contribute, take ownership and sustain the intervention. At the end of the community deliberations, key decision makers including district directors of health, politicians, chiefs, local health workers and non-governmental organisations (NGOs) were invited to discuss the priorities and solutions suggested by community members. This facilitated direct engagement and alignment of priorities between key decision makers and the community.

In our study, community members participated in problem solving and taking ownership of the interventions with minimal outside support [1, 2]. This community-centred method is in sharp contrast to vertical approaches in which solutions to community problems are externally determined and imposed on the community. Top-down approaches have failed sustainability tests and can often represent a cost to both the donors and the target community, with very minimal programme impact [2, 5]. The UNDP developed the CC approach, which emphasises community engagement as critical to addressing perceived issues through solutions deemed feasible by the community itself, on the premise that community members are the ones affected and should be the most motivated and engaged throughout the process [1, 2]. This CC approach encourages the use of available resources and community ownership through a shared perspective towards finding responsibility for proposing and implementing solutions to challenges. This results in practical and realistic suggestions rather than externally funded solutions that might prove difficult to sustain [2]. It is thus clear from our experience that similar problems may result in unique approaches to solutions if contextual community factors and preferences are considered.

The conversations functioned as an advocacy platform for large projects such as the construction of mother’s shelters and outreach health posts that would require the engagement of district health officials willing to undertake these projects in partnership with the community. In our experience, not every project identified as a top priority was achievable in light of the set criteria for support; therefore, in some communities, feasible projects were lower in priority. This addition of predetermined criteria for solutions was a modification of the CC methodology. It adds important value because not every community priority may be realistically addressed, and thus each community learned to critically evaluate the feasibility and sustainability of the proposed solutions to enhance their sustainability. It remains to be seen whether selected projects, with implementation, will be sustainable and achieve the desired long-term goals.

To address a general lack of knowledge about safe motherhood and family planning across the three districts, all communities proposed sensitisation campaigns using already existing community groups. Other problems unique to particular communities required specific interventions, such as constructing or renovating a mother’s shelter, buying motorised bicycle ambulances, and supporting existing women’s income-generating cooperative groups (data not shown). This variety of solutions showed that uniform approaches across communities are likely to be impractical [7].

Engaging communities often requires a form of “social contract” that defines clear roles and contributions from all parties [8, 9]. In this study, both the community and the project obligations were used to define a “social contract” agreed to by the community members that responsible parties can be held accountable through local leaders. Whether this approach will lead to improvement in targeted services has yet to be determined, but community engagement is certainly known to be a critical ingredient to encouraging the uptake and sustainability of community health interventions [3, 10–12].

We found evidence of compelling interactions between individual issues and the others, making it prudent to address these challenges collectively rather than one at a time [13]. We noted patterns of interconnected themes across the components of safe motherhood, e.g., knowledge of family planning, antenatal attendance, and facility versus home delivery, as demonstrated through the fishbone analysis (Fig. 2). Generally, from the demand side, a lack of information and misconceptions precluded women from benefitting from available services. Community members were aware of family planning, but misconceptions and fears were barriers to the uptake of modern contraceptives. Many participants felt that family planning could lead to promiscuity, cancer or infertility. This belief was stronger in rural sites than in peri-urban sites. Such fears have previously been reported in the maternal health literature from across sub-Saharan Africa [14, 15]. This differential uptake of contraceptive methods by region results in higher overall fertility rates in rural compared to urban areas, as reported by the Zambia Demographic and Health survey (DHS 2014) [4].

Participants’ lack of knowledge was also reflected in misconceptions regarding the optimal time to begin ANC (data not shown). Even when women were aware of the recommendations, they were limited to attending only a few visits due to the long distance between their homes and the health centres. Many women noted a preference to report later in pregnancy so that they could attend ANC visits fewer times (data not shown). There were also beliefs about the need to hide the pregnancy to avoid the risk of witchcraft being used against the unborn baby (data not shown).

Mistreatment of women presenting to health facilities for ANC or delivery was a common and regrettable theme. The barriers identified by poorer women due to their lack of hygiene, lack of clothes, or inability to procure delivery supplies represent an opportunity for re-training providers on compassionate and equitable care regardless of staff shortages, inadequate resources, or stress and fatigue. Rectifying adversarial relationships between women and their ANC providers could drastically improve ANC uptake, compliance, facility delivery rates, and postnatal care access. Above all, ensuring positive patient-provider interactions could restore community faith in the benefits of government-run health care.

Men may be discouraged from accompanying their spouses to ANC, as it may be time-consuming and mean a full day out of town, including travelling and waiting time; furthermore, activities in the facilities may not be well oriented for the participation of men [14]. It will be interesting to note how male involvement changes in sites targeted by this project.

An important limitation of this project was that the interventions focused mainly on demand-side issues, with minimal interventions on the supply side. Health workers’ attitudes and work ethic could adversely affect any intervention. Advocacy seems to have been beneficial in some areas. One group successfully lobbied the District Medical Officer for a female midwife, and another for formal training for TBAs. Further qualitative work is needed to understand what may best address the supply side of the interaction between patients and care providers. Although supply issues are supposed to be addressed through reports to the district health officials, there were insufficient data and evidence to discuss whether these reports were delivered and acted upon by district health officials.

Another limitation of this work was the short time between the CCs and expected outcomes. The issues targeted in this project were largely behavioural, and behaviours evolve over a long time with complex interdependencies. To appreciably change behaviour, sustained interventions with repeated evaluations may be required over a longer period of time.

## Conclusion

We successfully applied the CC approach to explore maternal health challenges and propose corresponding solutions in three rural districts of Zambia. We demonstrated that communities are willing to engage in CCs around maternal and newborn health and to collaborate on feasible interventions. Future work to explore how CCs have facilitated action in these communities will be important. Further studies are needed to understand the potential of CCs to increase levels of health care uptake in the medium term and their impact on maternal and newborn morbidity and mortality in the long term.

## References

[1] Campbell C, Nhamo M, Scott K, Madanhire C, Nyamukapa C, Skovdal M, et al. The role of community conversations in facilitating local HIV competence: case study from rural Zimbabwe. BMC Public Health 2013;13:354. doi: 10.1186/1471-2458-13-354. PubMed PMID: 23590640; PubMed Central PMCID: PMCPMC3637528. 10.1186/1471-2458-13-354 PMC3637528 23590640

[2] Boren SW (2013). Convening community conversations that matter. N C Med J.

[3] Sullivan J, Siqueira CE (2009). Popular arts and education in community-based participatory research (CBPR): on the subtle craft of developing and enhancing channels for clear conversations among CBPR partners. New Solut.

[4] Central Statistical Ofiice (CSO):. Zambia Demographic and Health Survey. 2014 Sep. Report No.: 0039–3665 (Print)

[5] Schwartz R, Price A, Deber RB, Manson H, Scott F (2014). Hopes and realities of public health accountability policies. Healthcare Policy.

[6] McHugh ML (2012). Interrater reliability: the kappa statistic. Biochem Med (Zagreb).

[7] Kohler JC, Mackey TK, Ovtcharenko N (2014). Why the MDGs need good governance in pharmaceutical systems to promote global health. BMC Public Health.

[8] Gilliam F, Penovich PE, Eagan CA, Stern JM, Labiner DM, Onofrey M (2009). Conversations between community-based neurologists and patients with epilepsy: results of an observational linguistic study. Epilepsy Behav.

[9] Zhang X, Bloom G, Xu X, Chen L, Liang X, Wolcott SJ (2014). Advancing the application of systems thinking in health: managing rural China health system development in complex and dynamic contexts. Health Res Policy Syst.

[10] Lobchuk M, McClement S, Rigney M, Copeland A, Bayrampour H (2015). A qualitative analysis of “naturalistic” conversations in a peer-led online support community for lung cancer. Cancer Nurs.

[11] Matthiesen M, Froggatt K, Owen E, Ashton JR (2014). End-of-life conversations and care: an asset-based model for community engagement. BMJ Support Palliat Care.

[12] Middleton L, Uys L (2009). A social constructionist analysis of talk in episodes of psychiatric student nurses conversations with clients in community clinics. J Adv Nurs.

[13] Storeng KT (2014). The GAVI Alliance and the ‘gates approach’ to health system strengthening. Glob Public Health.

[14] Bawah AA, Akweongo P, Simmons R, Phillips JF (1999). Women’s fears and men’s anxieties: the impact of family planning on gender relations in northern Ghana. Stud Fam Plan.

[15] Krakowiak-Redd D, Ansong D, Otupiri E, Tran S, Klanderud D, Boakye I (2011). Family planning in a sub-district near Kumasi, Ghana: side effect fears, unintended pregnancies and misuse of a medication as emergency contraception. Afr J Reprod Health.

